# TRPM2 channel: A novel target for alleviating ischaemia‐reperfusion, chronic cerebral hypo‐perfusion and neonatal hypoxic‐ischaemic brain damage

**DOI:** 10.1111/jcmm.14679

**Published:** 2019-09-30

**Authors:** Chendi Mai, Harneet Mankoo, Linyu Wei, Xinfang An, Chaokun Li, Dongliang Li, Lin‐Hua Jiang

**Affiliations:** ^1^ Sino‐UK Joint Laboratory of Brian Function and Injury of Henan Province and Department of Physiology and Neurobiology Xinxiang Medical University Xinxiang China; ^2^ Sanquan College of Xinxiang Medical University Xinxiang China; ^3^ School of Biomedical Sciences Faculty of Biological Sciences University of Leeds Leeds UK; ^4^ Xinxiang Maternal and Child Health Care Hospital Xinxiang China

**Keywords:** brain damage, chronic cerebral hypo‐perfusion, ischaemia‐reperfusion, neonatal hypoxia‐ischaemia, reactive oxygen species, TRPM2 channel

## Abstract

The transient receptor potential melastatin‐related 2 (TRPM2) channel, a reactive oxygen species (ROS)‐sensitive cation channel, has been well recognized for being an important and common mechanism that confers the susceptibility to ROS‐induced cell death. An elevated level of ROS is a salient feature of ischaemia‐reperfusion, chronic cerebral hypo‐perfusion and neonatal hypoxia‐ischaemia. The TRPM2 channel is expressed in hippocampus, cortex and striatum, the brain regions that are critical for cognitive functions. In this review, we examine the recent studies that combine pharmacological and/or genetic interventions with using in vitro and in vivo models to demonstrate a crucial role of the TRPM2 channel in brain damage by ischaemia‐reperfusion, chronic cerebral hypo‐perfusion and neonatal hypoxic‐ischaemia. We also discuss the current understanding of the underlying TRPM2‐dependent cellular and molecular mechanisms. These new findings lead to the hypothesis of targeting the TRPM2 channel as a potential novel therapeutic strategy to alleviate brain damage and cognitive dysfunction caused by these conditions.

## 1 INTRODUCTION

Human brain is a well‐known for its high and non‐stop metabolic demand, and thus, it is part of the human body that is most vulnerable to structural and functional damage by deprivation or restriction of oxygen and/or glucose supply, which is known to occur under conditions such as ischaemic stroke, cardiac arrest, chronic cerebral hypo‐perfusion and neonatal hypoxia‐ischaemia. Ischaemic stroke, mainly caused by cerebral ischaemia and contributing to approximately 80% of all stroke cases, represents the major cause of death and the most common cause of chronic disability in adults.^1^, ^2^, ^3^ Prolonged ischaemia can result in severe or fatal damage to the brain, and the currently available emergency procedures with medications aim to reinstate the blood circulation as soon as possible following ischaemia. It has been documented in rodent models as well as in stroke patients that reperfusion following transient ischaemia (henceforth referred to ischaemia‐reperfusion) exacerbates or causes further brain damage. Such ischaemia‐reperfusion damage significantly contributes to development of cognitive dysfunction and other neurological deficits that compromise the ability of stroke survivors to live a normal life. Therapeutics treating ischaemia‐reperfusion brain damage is still none but urgently required, considering the unpredictable nature and rapidly increasing prevalence of ischaemic stroke in modern society. Chronic cerebral hypo‐perfusion is widespread in adult brains, which is induced or exacerbated by ageing and numerous other risk factors such as hypertension, obesity and depression, and can cause grey and white matter atrophy and results in cognitive dysfunction and pre‐disposition to age‐related neurodegenerative diseases such as Alzheimer's disease (AD).^4^, ^5^ In neonates, hypoxia‐ischaemia and related condition hypoxic‐ischaemic encephalopathy are the common factors for death and severe impairments in sensorimotor and cognitive function in later life.^6^, ^7^ Hypothermia is the only treatment currently available, but hypothermia alone is often insufficient to prevent all neonatal hypoxic‐ischaemic brain damage and associated neurological deficits. There is a growing interest for additional and more effective neuroprotective treatment for the high prevalence and poor long‐term outcomes of this condition.

Brain damage caused by ischaemia‐reperfusion, chronic cerebral hypo‐perfusion or hypoxia‐ischaemia is an immensely complicated process that ultimately drives the demise of neurons via engaging many different types of cells.^5^, ^7^, ^8^, ^9^, ^10^, ^11^ Elucidating the underlying cellular and molecular mechanisms can facilitate a better understanding of the damage process, identification of novel drug targets and development of new therapeutic strategies to alleviate the cognitive dysfunction associated with brain damage caused under these conditions. Oxidative stress or an elevated level of reactive oxygen species (ROS), due to excessive ROS generation and/or impaired antioxidant capacity, is a salient feature of ischaemia‐reperfusion, particularly during reperfusion when oxygen molecules, the substrate required for diverse ROS‐generating mechanisms, become available after ischaemia, and ROS is a well‐recognized factor inducing ischaemia‐reperfusion brain damage.^8^, ^9^, ^10^, ^11^, ^12^, ^13^ Oxidative stress is also well documented in chronic cerebral hypo‐perfusion and hypoxic‐ischaemic brain damage.^5^, ^7^ However, how oxidative stress causes ischaemia‐reperfusion and chronic cerebral hypo‐perfusion brain damage is not fully understood.

The transient receptor potential melastatin‐related 2 (TRPM2) ion channel has been recognized as a molecular mediator of ROS‐induced cell death in a variety of cell types.^14^, ^15^ In this article, we examine the in vitro and in vivo studies that support an important role for the TRPM2 channel in brain damage, as a result of ischaemia‐reperfusion, chronic cerebral hypo‐perfusion and neonatal hypoxia‐ischaemia. We also discuss the current understanding of the underlying cellular and molecular mechanisms and the emerging evidence favouring the hypothesis of targeting the TRPM2 channel as a therapeutic strategy to alleviate brain damage and associated cognitive dysfunction under these conditions.

## 2 TRPM2 CHANNEL AS A COMMON MOLECULAR MECHANISM MEDIATING ROS‐INDUCED CELL DEATH

The TRPM2 channel belongs to the superfamily of transient receptor potential (TRP) channels^15^, ^16^, ^17^, ^18^ and is a tetrameric Ca^2+^‐permeable non‐selective cation channel that is gated by intracellular ADP‐ribose (ADPR) and cyclic ADPR.^19^, ^20^, ^21^, ^22^, ^23^, ^24^, ^25^ Intracellular Ca^2+^ can bind to and activate the TRPM2 channels,^26^ and warm temperature (≥35°C) can also induce the TRPM2 channel opening independently of, and more often in synergy with, ADPR or cyclic ADPR.^27^, ^28^ The TRPM2 channel can be potently activated after exposure to pathologically relevant concentrations of ROS, which is thought to stimulate ADPR generation via poly(ADPR) polymerase (PARP), particularly PARP‐1, and poly(ADRP) glycohydrolase (PARG) in the nucleus, and also via NADase in the mitochondria.^15^ The TRPM2 channel is expressed in many different types of cells,^15^ and a large body of evidence has been accumulated, since two seminal studies reported at the beginning of this century,^29^, ^30^ that supports the TRPM2 channel as an important and widespread molecular mechanism conferring the susceptibility to cell death induced by ROS and also by a diversity of pathological factors that are known to induce ROS generation.^31^, ^32^, ^33^, ^34^, ^35^, ^36^, ^37^, ^38^, ^39^, ^40^, ^41^, ^42^, ^43^, ^44^

In the brain, the expression of the TRPM2 channel has been shown in a subset of hypothalamic neurons that acts as a heat sensor that contains fever response to protect overheating.^45^ Neuronal expression of the TRPM2 channel has been also documented in hippocampus,^38^, ^41^, ^46^, ^47^ cortex,^48^ striatum 49 and substantia nigra,^42^, ^50^ the brain regions that are critically involved in cognitive and other neurological functions. The expression of the TRPM2 channel has been also reported in astrocytes,^51^, ^52^ particularly microglial cells that are known as the brain‐resident macrophage cells.^37^, ^53^, ^54^ Furthermore, the TRPM2 channel is expressed in cerebrovascular endothelial cells^35^ and pericytes,^36^ which together with astrocytes form the blood‐brain barrier (BBB), and plays a vital role in the regulation of neurovascular functions. There is accumulating evidence to show an important role of the TRPM2 channel in neurodegenerative diseases. Amyloid‐β peptide‐induced ROS generation‐mediated activation of the TRPM2 channel results in synaptic loss and neuronal death in hippocampus,^38^, ^41^, ^55^ microglial cell activation and generation of proinflammatory mediators^37^, ^55^, ^56^ and impairments in the BBB and neurovascular function,^35^ supporting a critical role of the TRPM2 channel in the pathogenesis of AD.^57^

## 3 TRPM2 CHANNEL IN ISCHAEMIA‐REPERFUSION BRAIN DAMAGE

There is growing attention to the TRPM2 channel in brain damage due to abnormal or insufficient supply of oxygen and glucose. Studies by combining pharmacological and genetic interventions, particularly recent studies using transgenic TRPM2‐knockout (TRPM2‐KO) mice and cells derived from the TRPM2‐KO mice, with various in vitro and in vivo models have revealed an important role of the TRPM2 channel in brain damage following ischaemia‐reperfusion, chronic cerebral hypo‐perfusion and neonatal hypoxia‐ischaemia (Table 1). Oxygen‐glucose deprivation (OGD) followed by reoxygenation (ODG‐R) and supplementation with glucose of extracellular solutions, often artificial cerebrospinal fluid, is commonly used as an in vitro model of ischaemia‐reperfusion. Transient middle cerebral artery occlusion (MCAO) followed by reperfusion (MCAO‐R) is an in vivo model of focal cerebral ischaemia and closely mimics ischaemic stroke in humans. Transient cardiac arrest (CA) followed by resuscitation (CA‐R) or transient bilateral common carotid artery occlusion (BCCAO) followed by reperfusion (BCCAO‐R) are two frequently used in vivo models of global ischaemia‐reperfusion. Bilateral common carotid artery stenosis (BCAS) can be used to introduce chronic cerebral hypo‐perfusion. Restriction of the common carotid artery is part of the procedures inducing hypoxic‐ischaemic brain damage.

**Table 1:** TRPM2 channel in ischaemia‐reperfusion, chronic cerebral hypo‐perfusion and neonatal hypoxia‐ischaemia brain damage

Damage indicators	Models	Key observations	References
Neuronal death	OGD‐R	Neuronal death in cultured mouse cortical neurons was strongly inhibited by treatment with TRPM2 inhibitors before ischaemia and during ischaemia‐reperfusion. Neuronal death was significantly attenuated in cultured mouse cortical neurons infected with shRNA‐mediated knockdown of the TRPM2 expression.	58
	OGD‐R	Neuronal death in cultured mouse hippocampal neurons was markedly suppressed by treatment with TRPM2 inhibitors before ischaemia and during ischaemia‐reperfusion. Neuronal death in cultured mouse hippocampal neurons was also significantly attenuated by treatment with CTZ after reoxygenation. Neuronal death in cultured mouse hippocampal neurons was reduced by shRNA‐mediated knockdown of the TRPM2 expression.	59
	OGD‐R	Neuronal death in cultured mouse cortical neurons was reduced by TRPM2‐KO.	60
	OGD‐R	Neuronal death of CA1 pyramidal neurons in mouse hippocampal slices was prevented by TRPM2‐KO.	61
	MCAO‐R	Neuronal death in mice was significantly reduced by administration of CTZ after reperfusion.	64
	MCAO‐R	Neuronal death in the neocortex in mice was substantially attenuated by TRPM2‐KO.	63
	CP‐R	Neuronal death of CA1 pyramidal neurons in the hippocampus of mice was significantly lessened by injection of CTZ after resuscitation.	66
	BCCAO‐R	Neuronal death of CA1 pyramidal neurons in the hippocampus of mice was protected by TRPM2‐KO.	61
Infarction	MCAO‐R	Infarct volume in mice was significantly reduced by injections of CTZ after ischaemia and at the beginning of reperfusion. Infarct volume in the striatum in mice was significantly reduced by injection of lentivirus expressing TRPM2‐shRNA before ischaemia.	58
		Infarct volume in the ischaemia hemisphere in mice was significantly attenuated by TRPM2‐KO.	60, 62, 63
		Infarct volume in the ischaemia hemisphere was substantially alleviated by injection of CTZ after reperfusion in WT, but not TRPM2‐KO mice.	60
		Infarct volume in the ischaemia hemisphere was lessened by administration of tat‐M2NX prior to ischaemia in WT, but TRPM2‐KO mice. Infarct volume in the ischaemia hemisphere in mice was also lessened by administration of tat‐M2NX after reperfusion in adult and aged mice.	64
	MCAO	Infarct volume in mice after permanent ischaemia without reperfusion was not reduced by TRPM2‐KO.	62
	H‐I	Infarct volume in mouse puppies was reduced by TRPM2‐KO.	69
Atrophy	BCAS	White matter atrophy in mice was prevented by TRPM2‐KO.	68
Cognitive dysfunction	MCAO‐R	Neurological deficits in mice were significantly attenuated by TRPM2‐KO.	63
	BCCAO‐R	Impairments in learning and memory in mice were suppressed or prevented by TRPM2‐KO.	61
	BCAS	Impairments in cognitive function in mice were protected by TRPM2‐KO.	68
Sensorimotor dysfunction	H‐I	Sensorimotor dysfunction was mitigated in mouse puppies by TRPM2‐KO.	69

Abbreviations: BCAS, bilateral common carotid artery stenosis; BCCAO‐R, bilateral common carotid artery occlusion‐reperfusion; CA‐R, cardiac arrest‐resuscitation; CTZ, clotrimazole; H‐I, hypoxia‐ischaemia; MCAO‐R, middle cerebral artery occlusion‐reperfusion; ODG‐R, oxygen‐glucose deprivation; TRPM2‐KO, TRPM2‐knockout.

### 3.1 TRPM2 channel in delayed neuronal death induced by OGD‐R

Herson and colleagues among other research groups were the first to investigate the role of the TRPM2 channel in delayed neuronal death induced by ischaemia‐reperfusion in vitro.^58^, ^59^, ^60^ They measured the viability of cultured cortical and hippocampal neurons after exposure to OGD‐R, using 3‐(4,5‐dimethylthiazol‐2‐yl)‐2,5‐diphenyltetrazolium (MTT) assay. In both neuron preparations, neuronal death, as indicated by a reduction in the cell viability, was strongly attenuated by treatment, before ODG and during OGD‐R, with N‐(p‐amylcinnamoyl) anthranilic acid (ACA), clotrimazole (CTZ), flufenamic acid (FFA) or 2‐aminoethoxy diphenyl borate (2‐APB).^58^, ^59^ Even though these inhibitors are limited in their specificity to the TRPM2 channel,^15^ these results collectively suggest that the neuroprotection results from inhibition of the TRPM2 channel. Consistently, neuronal death was significantly reduced in cultured cortical and hippocampal neurons infected with lentivirus expressing TRPM2‐specific short hairpin RNA (TRPM2‐shRNA) to reduce the TRPM2 expression, prior to ODG‐R.^58^, ^59^ Interesting and therapeutically important is that delayed neuronal death induced by OGD‐R in cultured hippocampal neurons was inhibited by treatment with CTZ, 15 minutes after reoxygenation, and the inhibition was almost as effective as that by treatment with CTZ starting prior to OGD‐R.^58^ Furthermore, neuronal death in cultured cortical neurons from the TRPM2‐KO mice was noticeably lower than that in cultured cortical neurons from the WT mice.^60^ We examined the role of the TRPM2 channel in delayed neuronal death in mouse hippocampal slices as a result of exposure to OGD‐R, using propidium iodide (PI) staining assay, focusing on pyramidal neurons in the CA1 region of hippocampus,^61^ because these cells are well‐known for their high vulnerability to ischaemia‐reperfusion damage. Neuronal death was observed in hippocampal slices from the WT mice, which was strongly suppressed by TRPM2‐KO.^61^ These studies, using cultured neurons and brain slices in conjunction with pharmacological and genetic interventions, show that the TRPM2 channel plays a critical role in mediating delayed neuronal death following ischaemia‐reperfusion.

### 3.2 TRPM2 channel in brain damage by focal cerebral ischaemia‐reperfusion

Several groups have employed the MCAO‐R model to study the role of the TRPM2 channel in ischaemia‐reperfusion brain damage in mice that are related to ischaemic stroke in humans.^58^, ^60^, ^62^, ^63^ In the WT mice subjected to MCAO‐R, brain damage in the cortex and striatum as well as in the whole ischaemia brain hemisphere, determined by measuring the infarct volume after reperfusion, was considerably lessened by subcutaneous injections of CTZ (30 mg/kg) twice, immediately after ischaemia and also at the beginning of reperfusion.^60^ Such CTZ‐induced protective effect was absent in the TRPM2‐KO mice, indicating that CTZ protects against ischaemia‐reperfusion brain damage via inhibiting the TRPM2 channel. The striatal infarct volume was also significantly reduced in the mice injected with lentivirus expressing TRPM2‐specific shRNA into the striatum 2‐3 weeks before ischaemia.^58^ Three independent studies compared MCAO‐R induced brain damage in the WT and TRPM2‐KO mice, and these studies provide independent but consistent evidence to show that ischaemia‐reperfusion brain damage was significantly protected by TRPM2‐KO.^60^, ^62^, ^63^ Furthermore, one of the studies noted no difference in brain damage between the WT and TRPM2‐KO mice that were subjected to permanent ischaemia without reperfusion.^62^ Such an observation may be interpreted to indicate that TRPM2‐KO conferred no protection against ischaemia‐induced brain damage, or alternatively the protection was overwhelmed by the severe damage induced by prolonged ischaemia.^62^ It was also found in one of the studies that MCAO‐R induced neuronal death in the neocortex and neurological deficits were attenuated by TRPM2‐KO.^63^ A more recent study has reported that brain damage in the WT mice was significantly alleviated by administration of tat‐M2NX (20 mg/kg), a cell‐permeable peptide inhibitor of the TRPM2 channel, prior to MCAO and, importantly, such protection was lacking in the TRPM2‐KO mice, indicating that tat‐M2NX specifically inhibits the TRPM2 channel as intended.^64^ Such a protective effect was even persistent for several days after the initial ischaemia.^64^ Brain damage induced by MCAO‐R and determined 24 hours after reperfusion in aged mice (18‐20 months old) was also effectively protected by administration of tat‐M2NX 30 minutes after reperfusion.^64^ These findings suggest that the TRPM2 channel activation during reperfusion is critical in determining ischaemia‐reperfusion brain damage and provide the proof of concept that post‐ischaemia intervention of the TRPM2 channel during reperfusion is a promising strategy to alleviate ischaemic stroke damage brain.

### 3.3 TRPM2 channel in delayed neuronal death after global ischaemia‐reperfusion

The role of the TRPM2 channel in mediating delayed neuronal death has also been investigated in vivo, using the CP‐R model^65^ or the BCCAO‐R model^61^ to introduce global ischaemia‐reperfusion. Delayed neuronal death in the CA1 region of the hippocampus in the WT mice subjected to CP‐R was markedly reduced by subcutaneous injection of CTZ 30 minutes after resuscitation.^65^ Consistently, delayed neuronal death in the CA1 region of the hippocampus in the mice subjected to BCCAO‐R was protected by TRPM2‐KO.^61^ Furthermore, BCCAO‐R induced impairments in learning and memory, examined by novel habitation test and water maze test, were mitigated or prevented by TRPM2‐KO.^61^ Thus, these studies using different in vivo models consistently support an important role for the TRPM2 channel in mediating delayed neuronal death and cognitive dysfunction related to ischaemia‐reperfusion brain damage.

### 3.4 TRPM2 channel in ischaemia‐reperfusion brain damage is sexually dimorphic

It is known that ischaemia‐reperfusion brain damage exhibits strong sexual dimorphism in rodent models and stroke patients. Herson and colleagues studied, using in vitro and in vivo models, whether TRPM2‐dependent ischaemia‐reperfusion brain damage was also sexual dimorphic.^58^, ^59^, ^60^, ^64^, ^65^, ^66^ The TRPM2 channel expression or activity level was similar in cortical and hippocampal neurons from male and female mice. However, in contrast with the results described above from studies using male mice or neurons from male mice, delayed neuronal death induced in vitro in cortical and hippocampal neurons from female mice by OGD‐R, delayed neuronal death in vivo in female mice by CA‐R, or infarction induced in female mice by MCAO‐R, were not significantly reduced by treating with CTZ or tat‐M2NX, by reducing the TRPM2 expression using lentivirus expressing TRPM2‐specific shRNA, or by genetically deleting the TRPM2 expression. These results clearly indicate strong sexual dimorphism with respect to the role of the TRPM2 channel in mediating ischaemia‐reperfusion brain damage.

Sex steroids and their signalling pathways have been investigated for their potential role in determining the dimorphic outcomes of targeting the TRPM2 channel to protect against brain damage induced by MCAO‐R.^58^, ^60^, ^67^ CTZ‐induced neuroprotection was lost in the male mice that were prior castrated to remove endogenous androgens, but was restored by implanting dihydrotestosterone (DHT) in the castrated male mice, leading to the suggestion of involvement for the androgen receptor signalling.^60^ However, a subsequent study showed that administration of CTZ resulted in no significant protection against ischaemia‐reperfusion brain damage in the female mice that were hormonally intact but subjected to ovariectomization.^67^ In addition, implanting DHT in the female mice, even at an increased dose, afforded no neuroprotection, suggesting that circulating sex steroids such as androgen are insufficiently responsible for sexual difference.^67^

## 4 TRPM2 CHANNEL IN CHRONIC CEREBRAL HYPO‐PERFUSION BRAIN DAMAGE

Kaneko and colleagues have recently examined the role of the TRPM2 channel in mediating chronic cerebral hypo‐perfusion brain damage induced by BCAS in male mice.^68^ Significant white matter atrophy and, consistently, impairment in cognitive functions, examined using the Y‐maze test, were observed in mice after they were subjected to hypo‐perfusion for 28 days. However, there was neither detectable neuronal death in the hippocampus and cortex nor impairment in the BBB function in the grey matter. Both white matter damage and cognitive dysfunction were prevented by TRPM2‐KO.^68^ These findings therefore support that the TRPM2 channel also plays a significant role in mediating chronic cerebral hypo‐perfusion brain damage. Male mice were used in this study, and it is unclear whether the protection against chronic cerebral hypo‐perfusion brain damage by inhibiting the TRPM2 channel is also sex‐dependent.

## 5 TRPM2 CHANNEL IN NEONATAL HYPOXIC‐ISCHAEMIC BRAIN DAMAGE

Sun and colleagues have recently explored the role of the TRPM2 channel in neonatal hypoxic‐ischaemic brain damage in postnatal day 7 pups induced by ligating the right common carotid artery and exposing to reduced oxygen level.^69^ Brain damage, examined 24 hours or 7 days after hypoxia‐ischaemia, was significantly reduced in the heterozygous and homozygous TRPM2‐KO pups compared to that in the WT pups. Sensorimotor dysfunction, examined using the cliff avoidance test and geotaxis test 7 days after hypoxia‐ischaemia, was substantially mitigated in the heterozygous and homozygous TRPM2‐KO mice. These results indicate that the TRPM2 channel plays an important role in mediating neonatal hypoxic‐ischaemic brain damage.

## 6 CELLULAR AND MOLECULAR MECHANISMS MEDIATING BRAIN DAMAGE

**Figure 1:**
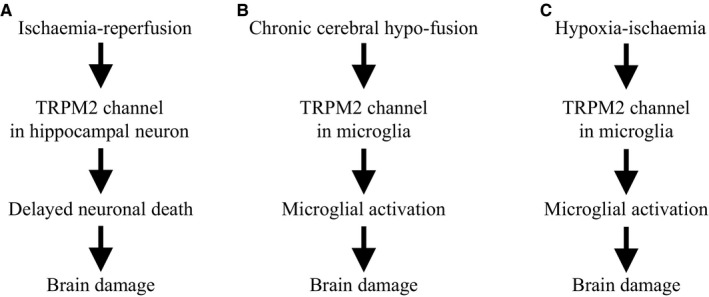
TRPM2‐dependent cellular mechanisms for brain damage. Elevated generation of reactive oxygen species (ROS) is a common feature of ischaemia‐reperfusion, chronic cerebral hypo‐fusion and neonatal hypoxia‐ischaemia. A, Activation of the TRPM2 channel in hippocampal neurons mediates delayed neuronal cell death, contributing to ischaemia‐reperfusion or ischaemic stroke brain damage. B‐C, Activation of the TRPM2 channel in microglia initiates microglial activation in chronic cerebral hypo‐fusion and neonatal ischaemia‐hypoxic brain damage. TRPM2‐mediated infiltration of peripheral immune cells and astrocyte activation also contribute to brain damage by ischaemia‐reperfusion and neonatal hypoxia‐ischaemia, respectively (not depicted). See text for more details

Neurons are the key player in determining neurological functions of the brain and, in the simplest term, loss of neurons and/or their functions is directly responsible for cognitive dysfunction and other neurological deficits. Hippocampal, cortical and striatal neurons are known to be critical for mediating cognitive functions, particularly learning and memory. Studies using cultured hippocampal, cortical and striatal neurons in combination with pharmacological and genetic interventions provide clear evidence to show that activation of the neuronal TRPM2 channel in mediating neuronal death induced by ROS and under pathological conditions that are known to generate ROS.^38^, ^41^, ^42^, ^48^, ^49^, ^55^, ^59^ Brain damage by ischaemia‐reperfusion, chronic cerebral hypo‐perfusion and neonatal hypoxic‐ischaemia, as discussed above, is highly complicated, engaging other types of cells in the brain, particularly microglial cells. Aberrant microglial cell activation can lead to excessive generation of neurotoxic proinflammatory mediators and neuroinflammation, which represents a contributing factor in a wide spectrum of brain pathologies, including ischaemic stroke, traumatic brain damage, AD, Parkinson's disease, multiple sclerosis and psychiatric disorders. The TRPM2 channel is highly expressed in microglial cells. There is compelling evidence from recent studies that supports an important role for the TRPM2 channel in mediating microglial cell activation and generation of proinflammatory mediators and neuroinflammation in response to stimulation of oxidative stress or stimuli that known to induce ROS generation such as amyloid‐β peptides.^37^, ^56^, ^57^ It has been also shown that TRPM2‐mediated activation of microglial cells contributes to chronic cerebral hypo‐perfusion brain damage^68^ and activation of microglia and astrocytes in neonatal hypoxic‐ischaemic brain damage.^69^ It remains unknown regarding the role of TRPM2‐dependent microglial activation and neuroinflammation in ischaemia‐reperfusion‐induced delayed neuronal death. However, there is evidence to indicate a significant role of the TRPM2 channel in peripheral immune cells in mediating their activation and infiltration into the brain to worsen ischaemia‐reperfusion brain damage.^63^ The major cellular mechanisms currently known to mediate brain damage by ischaemic stroke, chronic cerebral hypo‐perfusion and neonatal hypoxia‐ischaemia are summarized in Figure 1.

**Figure 2:**
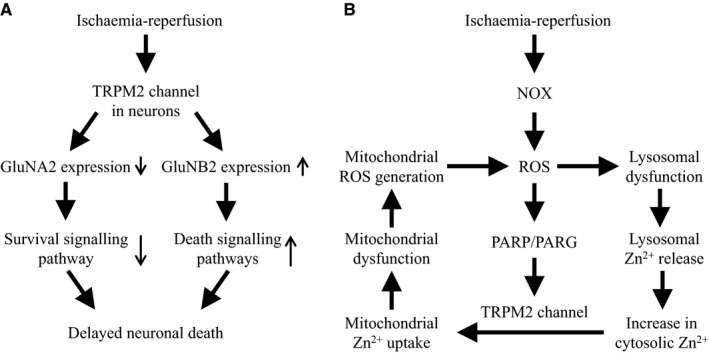
TRPM2‐dependent molecular mechanisms for delayed neuronal death. Two distinctive TRPM2‐mediated molecular mechanisms for delayed neuronal death leading to ischaemia‐reperfusion brain damage have been proposed. A, Elevated generation of reactive oxygen species (ROS) during ischaemia‐reperfusion and subsequent activation of the TRPM2 channel in hippocampal neurons induce down‐regulation of the GluNA2‐containing NMDAR‐mediated survival signalling pathway and up‐regulation of the GluNB2‐containing NMDAR‐mediated death‐promoting signalling pathways, resulting in neuronal death. B, Elevated ROS during reperfusion following transient ischaemia stimulates NADPH‐dependent oxidases (NOX)‐mediated ROS generation. ROS causes lysosomal loss and dysfunction and release of Zn^2+^, elevating the cytosolic Zn^2+^ level. ROS also induces activation of the TRPM2 channel in the mitochondria as well as on the cell surface via promoting ADPR generation catalysed by poly(ADPR) polymerase (PARP) and poly(ADPR) glycohydrolase (PARG) in the nucleus. Activation of the TRPM2 channel in the mitochondria increases mitochondrial uptake of Zn^2+^ that triggers mitochondrial loss and dysfunction and mitochondrial ROS generation. Therefore, activation of the TRPM2 channel sets in motion a positive feedback mechanism ultimately drives lysosomal and mitochondrial dysfunction and neuronal death

It is less well understood with respect to the molecular mechanisms by which the TRPM2 channel is activated in immunocompetent cells, particularly microglial cell activation. In neurons, two distinctive molecular mechanisms, by which the TRPM2 channel mediates delayed neuronal death contributing to ischaemia‐reperfusion brain damage, have been proposed.^61^, ^62^ It is worth pointing out that these mechanisms are not mutually exclusive. The N‐methyl‐D‐aspartate (NMDA) subfamily of the glutamate receptors (NMDAR) is well documented for their importance in neuronal survival and death.^70^ More specifically, the GluN2A‐containing NMDAR‐mediated signalling pathways are neuroprotective and, by contrast, the GluN2B‐containing NMDAR‐mediated signalling pathways promote neuronal death. TRPM2‐KO led to elevated synaptic excitability in hippocampal CA1 neurons, which was abolished by antagonising the GluN2A‐containg NMDAR. Consistently, TRPM2‐KO selectively up‐regulated the GluN2A expression and, at the same time, down‐regulated the GluN2B expression in hippocampal neurons. These observations prompted the proposal that activation of the TRPM2 channel suppresses the GluN2A‐mediated survival signalling pathways and enhances the GluN2B‐ mediated death signalling pathways, leading to neuronal death (Figure 2A).^62^

It is long known that an aberrant increase in the [Ca^2+^]_i_ after transient ischaemia is recognized to be critical in inducing delayed neuronal death.^10^ The TRPM2 channel is a non‐selective cationic channel with a substantial permeability to Ca^2+^. Evidence exists that TRPM2‐mediated increase in the [Ca^2+^]_i_ induces neuronal death in cultured hippocampal, striatal and cortical neurons.^38^, ^42^, ^48^ Zn,^2+^ a trace metal ion being an important enzyme co‐factor, is known for its neurotoxicity. There is compelling evidence to show that an increase in the [Zn^2+^]_i_ during reperfusion, which was attributed as part of the increase in the [Ca^2+^]_i_, is important in inducing delayed neuronal death.^43^, ^71^, ^72^ As was observed in both mouse brain slices subjected to OGD‐R and mice subjected to BCCAO‐R, the increase in the [Zn^2+^]_i_ and delayed neuronal death in hippocampal pyramidal neurons were reduced by TRPM2‐KO.^61^ Single‐cell imaging revealed that the increase in the [Zn^2+^]_i_ during OGD was the same between the WT and TRPM2‐KO hippocampal neurons, and that the [Zn^2+^]_i_ remained persistently high in the WT neurons upon reperfusion but declined rapidly in the TRPM2‐KO neurons, indicating an exclusive role for the TRPM2 channel in post‐ischaemic increase in the [Zn^2+^]_i_.^61^ Zn^2+^ is known to inhibit mitochondrial function and stimulate mitochondrial ROS generation.^72^ ROS generation in the hippocampus after BCCAO‐R was strongly attenuated in the TRPM2‐KO mice.^61^ These observations prompt the hypothesis that post‐ischaemic activation of the TRPM2 channel triggers a vicious cycle, composed of an increase in the [Zn^2+^]_i_, mitochondrial dysfunction and mitochondrial ROS generation, that ultimately drives neuronal death.^61^ In a recent study examining human SH‐SY5Y neuroblastoma cells, we have shown that H_2_O_2_ induced neuronal cell death with a significant delay, requiring the TRPM2 channel activation and TRPM2‐dependent increase in the [Zn^2+^]_i_.^73^ We have further used such a neuronal cell model to gather strong evidence to suggest that ROS‐induced activation of the TRPM2 channel sets in motion a vicious positive feedback signalling mechanism for delayed neuronal death (Figure 2B).^73^

## 7 CONCLUDING REMARKS AND PERSPECTIVE

Here, we provide an overview of the recent literature that demonstrates the important role of the TRPM2 channel in brain damage caused by altered or insufficient supply due to ischaemia‐reperfusion, chronic cerebral hypo‐perfusion and hypoxia‐ischaemia. Studies also begin to gather encouraging evidence to support the hypothesis that the TRPM2 channel is an attractive novel target for development of therapeutics mitigating brain damage and cognitive dysfunction associated with ischaemic stroke. From the discussion above, it is clear that more investigations are required to provide integrated insights into the TRPM2‐dependent cellular and molecular mechanisms that contribute to brain damage as a result of ischaemia‐reperfusion, chronic cerebral hypo‐perfusion and hypoxia‐ischaemia. A better understanding of these damage processes at the molecular levels is critical for developing feasible therapeutic strategies of targeting the TRPM2 channel to alleviate neurological dysfunction accompanying brain damage under such conditions.

## CONFLICTS OF INTEREST

The authors declare no conflict of interest.

## AUTHOR CONTRIBUTION

All the authors participated in analysing and discussing the literature, commenting on and approving the manuscript. L‐HJ supervised the research, led the discussion, wrote and revised the manuscript.
